# Phenolic content, antioxidant and antimicrobial activities evaluation and relationship of commercial spices in the lebanese market

**DOI:** 10.1186/s13065-023-01074-2

**Published:** 2023-11-20

**Authors:** Youssef El Rayess, Lea Nehme, Chantal Ghanem, Marc El Beyrouthy, Carmen Sadaka, Samar Azzi-Achkouty, Nancy Nehme, Eda Sönmez Gürer, Javad Sharifi-Rad

**Affiliations:** 1 Department of Agriculture and Food Engineering, School of Engineering, Holy Spirit University of Kaslik (USEK), Jounieh, Lebanon; 2 Lebanese Agricultural Research Institute, Fanar Station, Fanar, Lebanon; 3 Madison, Wisconsin USA; 4 Faculty of Agricultural Engineering and Veterinary Medicine, Lebanese University, Dekwaneh, Lebanon; 5 Faculty of Pharmacy, Department of Pharmacognosy, Sivas Cumhuriyet University, Sivas, Turkey; 6 Facultad de Medicina, Universidad del Azuay, Cuenca, Ecuador

**Keywords:** Spices, Phenolic content, Antioxidant activity, Antimicrobial activity, Lebanese market

## Abstract

Lebanese cuisine is renowned for its distinctive flavours and vibrant aromas. In Lebanese cuisine, spices are not just used for their flavour; they are also valued for their medicinal properties. This study consists of evaluating and comparing the total phenolic content and the antioxidant capacity of 21 samples of spices used in the Lebanese daily diet, such as cinnamon, allspice, coriander, cloves, etc. and the mixtures prepared in well-defined proportions. Several solvents were tested for the extraction of the phenolic compounds from spices, and the water and ethanol (v/v) mixture were retained for this study. Results showed that clove presented the highest polyphenol content (173.7 ± 2.98 mg Gallic Acid Equivalent (GAE)/g Dry Matter (DM)) and the highest antioxidant capacity by ABTS test (4875.68 ± 480.40 µmol trolox / g DM). and DPPH test (85.84 ± 0.5%). The examination of the results showed a positive significant correlation between the polyphenol contents and the antioxidant activity of the spices. The antimicrobial activity tested by the broth microdilution method was determined against *Escherichia coli*, *Listeria monocytogenes*, *Staphylococcus aureus* and *Salmonella* Enteritidis. The results showed high antimicrobial activity manifested by low value of minimum inhibitory concentration (MIC) (MIC < 2.4 µg/mL) for cinnamon, turmeric, white pepper, red pepper, allspice, clove and nutmeg. In conclusion, spices used in Lebanese cuisine, such as clove, cinnamon, allspice and spices, were rich in phenolic compounds and presented important potential health benefits.

## Introduction

An oxidative/antioxidant imbalance called oxidative stress is observed when the activity of free radicals exceeds the body’s self-defence capacity, leading to irreversible cell damage, cardiovascular disease, cancer, accelerated ageing, pulmonary edema, etc. [1].

Subsequently, an adequate intake of antioxidants is required to avoid or delay the severe consequences of oxidative stress. The antioxidant activity is summarised by various possible mechanisms such as free radical scavenging activity, metal ion chelating activity, inhibition of prooxidant enzymes, and activation of antioxidant enzymes to suppress, delay or prevent the oxidation process or its propagation [2].

Phenolic compounds or polyphenols are the most common antioxidants in nature [3]. These are secondary metabolites of plants [4] and represent a tremendous potential source of therapeutic agents [2]. The major sources of phenolic compounds are fruits, tea, wines and spices. The major classes of phenolic compounds are phenolic acids, flavonoids, tannins, coumarins, lignans, curcuminoids and stilbenes [5].

In addition to their culinary application, spices have been the subject of recent research due to their antioxidant properties. They are rich in antioxidant metabolites that can be used to protect against the effects of oxidative stress [6, 7]. Halvorsen et al., [8] have shown that spices, rich in antioxidants, are among the most antioxidant-containing foods per 100 g serving. The maximum concentration of total phenolic compounds can reach approximately 250 mg gallic acid equivalent/g dry matter with a maximum antioxidant capacity of 2000 µmol trolox / g dry matter [9, 10]. The major phenolic compounds and their health benefits were recently reviewed by Sing and Yadav [11]. Eugenol mainly occurs in clove, cinnamon, turmeric and nutmeg [12]. The most found phenolic compounds in black pepper and cinnamon are the phenolic acids [11]. Rhaponticin, stigmasterol and carvacrol are the main phenolic compounds present in fenugreek, coriander and caraway respectively [13–15].

Food poisoning is still a worldwide concern affecting consumers and the food industry. Since ancient times, spices have been added to food, not only as flavouring agents but also to prevent food spoilage and deterioration and to extend the shelf-life of foods. The literature contains many publications reporting the antimicrobial activity of spices and herbs. The natural bioactive compounds of spices belonging to the flavonoids, phenolic acids, aldehydes, monoterpenes and alkaloids have shown antimicrobial activities against food-relevant bacteria, including *E. coli*, *Listeria*, *Staphylococcus* and *Salmonella* [16, 17].

Lebanese cuisine is a symphony of different cultural heritages, and spices are essential ingredients of this cuisine. In the Lebanese market, spices like cinnamon, black pepper, etc., can be mixed individually for different uses, such as seven spices, chicken spices, etc.

Up to this point, numerous investigations have explored the antioxidant and antimicrobial properties of various spices across the globe, employing diverse analytical approaches. However, these studies have yet to be conducted on Lebanese spices, specifically focusing on the spice blends commonly employed in everyday culinary practices. Consequently, the aims of this research are: (i) to assess and compare the phenolic content, the antioxidant capacity and the antimicrobial activity of spices available in the Lebanese market; (ii) to determine the correlation between antioxidant activity and phenolic content.

## Materials and methods

### Spices

To carry out this study, 21 spice samples were obtained from the Lebanese agro-food industry, Aoun Food Co, one of the biggest spice suppliers in Lebanon. All spice samples were collected as a dried powder. Table 1 shows the spices used and their origin, while Table 2 presents the spice mixture and composition.

**Table 1:** Individual spices used in this study and their origin

Spices	Origin
Cinnamon: *Cinnamomum zeylanicum*	Vietnam
Coriander: *Coriandrum sativum*	Lebanon
Cumin: *Cuminum cyminum*	Sri-Lanka
Turmeric: *Curcuma longa*	India
Fenugreek: *Trigonella foenum-graecum*	India
Ginger: *Zingiber officinale*	India
Clove: *Syzygium aromaticum*	Sri-Lanka
Nutmeg: *Myristica fragrans*	Sri-Lanka
Red pepper: *Capsicum annuum*	India
White pepper: *Piper nigrum*	Vietnam
Allspice: *Pimenta dioica*	Mexico
Black pepper: *Piper nigrum*	Mexico
Caraway: *Carum carvi*	Egypt

**Table 2:** Name of spice mixtures and their composition

Mixture name	Spice composition
Sausage spices	Allspice, cinnamon, ginger, black pepper, clove, nutmeg, white pepper, coriander, fenugreek, salt
Taouk spices	White pepper, garlic powder, nutmeg, red pepper, ginger, salt
Seven spices	Allspice, cinnamon, ginger, black pepper, nutmeg, clove, coriander
Moghrabia spices	Cumin, anise, allspice, cinnamon, nutmeg, clove, cumin
Chicken Chawarma spices	Ginger, nutmeg, white pepper, garlic powder, salt
Meat Chawarma spices	Allspice, cinnamon, ginger, black pepper, nutmeg, clove, coriander, white pepper, salt
Siyadiyeh spices	Allspice, cinnamon, cumin, white pepper, black pepper, coriander, nutmeg, salt
Falafel spices	Coriander, allspice, cumin, cinnamon, black pepper, salt

### Extraction of polyphenols

The most used solvents in the extraction of phenolic compounds are ethanol and methanol. However, there is no standard method for extraction. Thus, in order to optimise the extraction of phenolic compounds, four mixtures of solvents were studied on one spice (Cinnamon): 100% ethanol, 100% methanol, 50% ethanol + 50% water and 50% methanol + 50% water.

For the extraction and analysis of the active principles in the spice samples, 200 mg of each spice was weighed into a falcon tube and vortexed with 2 ml of solvent for 10 min. Then, the falcon tubes were immersed in an ultrasonic bath for 2 min and centrifuged at 4 °C for 5 min at 4000 rpm. The supernatant was stored at 4 °C. These steps were repeated twice; thus, the total supernatant volume was 4 ml [18]. All extractions were made in triplicate.

### Total polyphenol determination

The total polyphenol content of the supernatants was determined spectrophotometrically at 760 nm (THERMO HEλIOS α) after the reaction with Folin-Ciocalteu reagent (Sigma-Aldrich, 1,090,010,500) according to the method described by Dawra et al. [19]. The results were expressed as gallic acid equivalents (mg GAE) / g of dry matter (DM). A calibration curve was prepared with gallic acid standard solutions with concentrations ranging from 0 to 2000 mg/l (R^2^ = 0.999).

### Scavenging of ABTS radical cation assay

The antioxidant activity was determined by measuring the ability to scavenge 2,2′-azinobis (3-ethylbenzothiazline-6-sulfonic acid) (ABTS) (Sigma-Aldrich, A1888-5G) radical cation produced by the oxidation of ABTS with manganese dioxide as previously described by Re et al. [20]. The absorbance was measured directly after 6 min at 734 nm and at 25 °C. The antioxidant capacity was expressed as µmol Trolox/g DM.

### DPPH radical-scavenging activity

The DPPH radical-scavenging activity (Sigma-Aldrich, D9132) was determined using the method proposed by Masuda et al. [21]. After incubation in the dark for 20 min at room temperature, the absorbances were read at 517 nm using a spectrophotometer (THERMO HEλIOS α).

The inhibition of the DPPH radical was calculated by the following formula (1):

1 $${\text{DPPH scavenging effect }}\left( \% \right)\, = \,\left[ {{A_0}-{A_1}/{A_0}} \right]\, \times \,100$$

A_0_ and A_1_ are the absorbance at 30 min of the control and the sample, respectively.

### Antimicrobial activity

The broth microdilution MIC test was used to test the antimicrobial activity of the spices’ extracts. All the bacterial strains used in this study were generously provided by the Lebanese Agriculture Research Institute (LARI). The studied bacteria were *Escherichia coli* ATCC 8739, *Staphylococcus aureus* ATCC 25,923, *Listeria monocytogenes* ATCC 19,115 and *Salmonella* Enteritidis isolated from broilers samples collected from Lebanese slaughterhouses. A bacterial suspension of each bacterial strain was prepared in a Mueller Hinton Broth (MHB) (Merck KGaA) at a concentration of 2.10^8^ CFU/ml (0.5 McFarland standard) [22]. The minimum inhibitory concentration (MIC) values of the spice’s extracts were determined by serial dilution in a 96-well microplate. Each well first contained 100 µL of MHB. The dried extracts were dissolved in pure DMSO (Sigma-Aldrich, 472,301) to a 5 mg/ml concentration. Afterwards, 100 µL of the latter were placed in the first well, and a serial dilution was conducted in order to obtain the following concentrations in each row: 2500, 1250, 625, 312.5, 156, 78.125, 39.06, 19.5, 9.76, 4.88 and 2.4 µg/ml. Next, 100 µL of each bacterial strain tested were added to the extract solutions. Therefore, each well’s initial bacterial concentration was adjusted to 108 CFU/ml. The negative control was composed of 100 µL of DMSO and 100 µL of the bacterial strain tested, while the positive control contained 100 µL of MHB and 100 µL of the bacterial strain. Sterile DMSO served as the negative control. The absorbance at 620 nm was measured at time 0 h and after an overnight incubation at 37^°^C (24 h) using a Multiskan Sky Microplate Spectrophotometer (Thermo Fisher Scientific, USA).

### Statistical analysis

All experiments were carried out in triplicate. Analysis of variance (ANOVA) and Tukey’s honestly significant difference (HSD) test were used for mean separation, with a significant level of 95% (*p* ˂ 0.05). These statistical analyses and Pearson correlation were conducted using Xlstat software (2014).

## Results and discussion

### Optimisation of polyphenol extraction

Phenolic compounds can be easily isolated from food by extraction with organic solvents. However, the extraction procedure is influenced by the chemical nature of the solvent. For this purpose, the extraction step has been optimised, and four solvents (100% ethanol, 100% methanol, ethanol + water (v/v) and methanol + water (v/v)) have been tested.

**Fig. 1:**
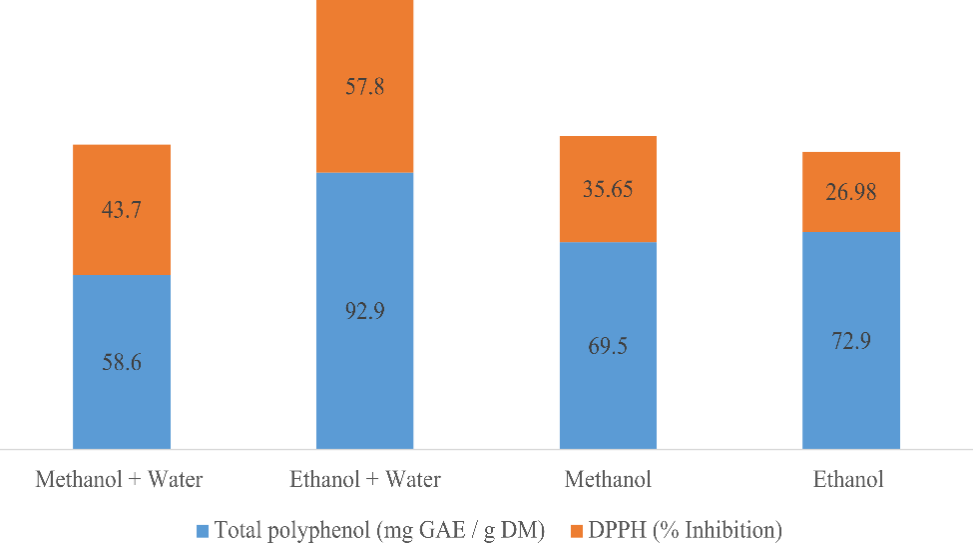
Total polyphenol and the inhibition % of cinnamon supernatants were extracted with different solvents

Figure 1 shows the content of total polyphenols and the percentage of inhibition of cinnamon supernatants extracted with the four solvents. The extract prepared with water and ethanol (v/v) presented the highest level of total polyphenols (92.9 mg GAE / g DM), while the one obtained with methanol and water (v/v) presented the lowest one (58.6 mg GAE / g DM). Also, the same mixture of solvent presented the highest antioxidant activity (57.8% of inhibition). This might be due to the binary solvent extraction of the ethanol–water system, which effectively extracted more hydrophilic and lipophilic phytochemicals than the single solvent system.

Thus, since the highest polyphenol content and antioxidant activity were exhibited when using the mixed solvent water + ethanol (v/v), this solvent was subsequently used for the extraction of the various samples of spices to be analysed.

### Total phenolic contents and antioxidant activity

**Fig. 2:**
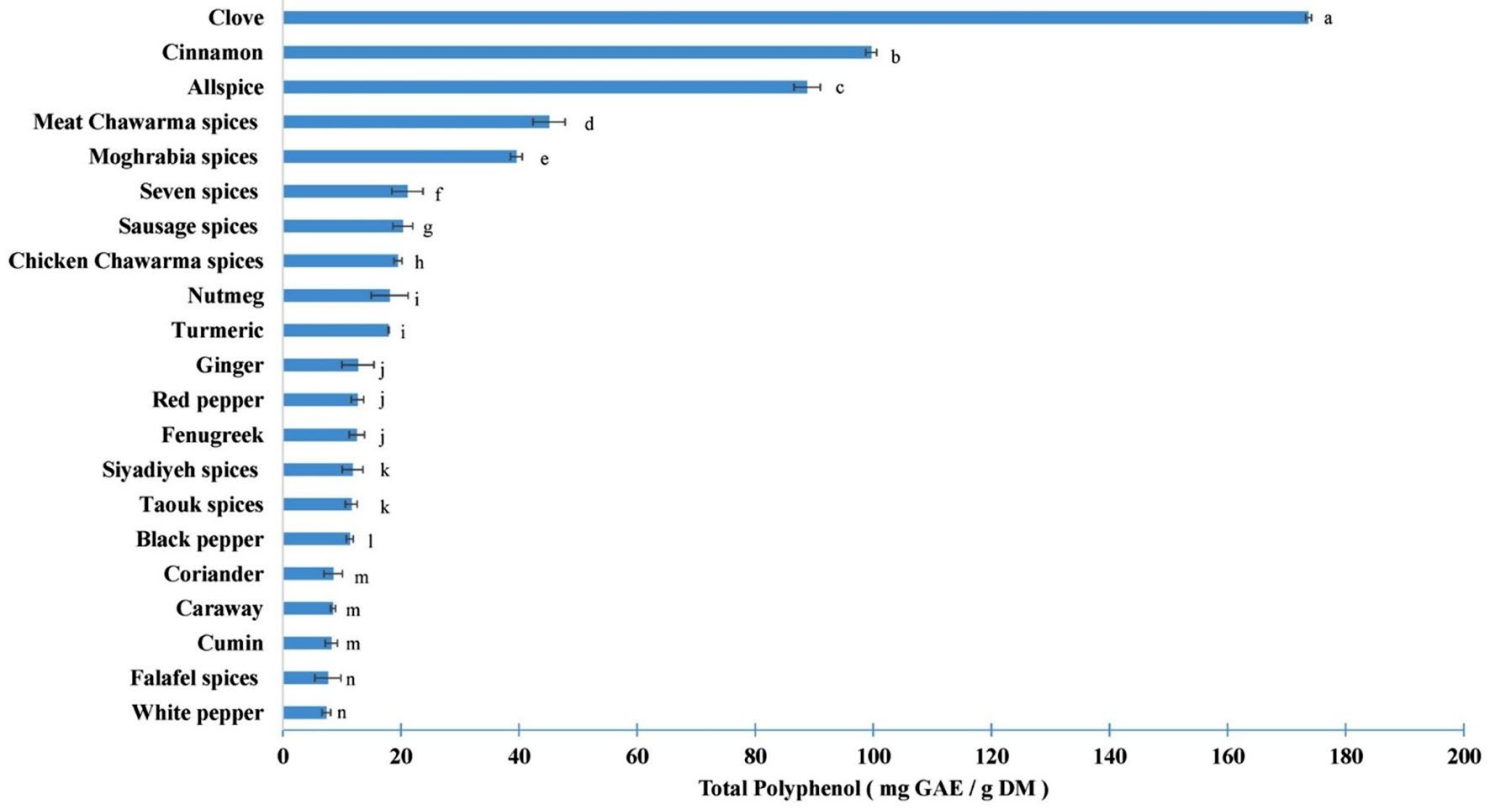
Total phenolic content of the different spices’ samples. Mean (n = 3). Error bars represent standard deviation. Different letters indicate significant differences at p < 0.05

Results from the quantitative determination of total phenolics are shown in Fig. 2. All the calculations were done using the standard equation obtained from the standard calibration curve of gallic acid (R^2^ = 0.9968).

The results showed a significant difference in the total polyphenol contents among the various samples. Clove exhibited the highest total polyphenol concentration, establishing itself as the most polyphenol-rich spice. Similarly, extracts of cinnamon and allspice also displayed a notable abundance in polyphenol content. In contrast, white pepper, cumin, caraway and coriander exhibited the lowest total polyphenol content. As for the spice mixtures, meat chawarma and moghrabia spices had the highest polyphenol content, owing to their rich composition of spices with high total polyphenol content such as clove, cinnamon and allspice. Conversely, falafel and taouk spices exhibited the lowest phenolic content.

When comparing the results of the current study to those obtained in other studies, both similarities and differences are evident, depending on the specific study. Table 3 provides a summary of the results obtained in other studies.

**Table 3:** Comparison of the total polyphenol content with other studies from the literature

		Total Polyphenol Content (mg GAE/g DM)			
**Spices / References**	Total polyphenol content	Shan et al. [23]	Khatun et al. [9]	Lu et al. [24]	Przygodzka et al. [18]
**White pepper**	7.37 ± 0.37	7.8 ± 0.04	0.93 ± 0.08	3.53 ± 0.02	3.2
**Cumin**	8.23 ± 0.28	2.3 ± 0.05	8.5 ± 0.2	9 ± 0.15	
**Caraway**	8.52 ± 0.2	6.1 ± 0.17			
**Coriander**	8.54 ± 0.28	8.8 ± 0.07	4.67 ± 0.4		2.2
**Black pepper**	11.34 ± 0.45	3 ± 0.05	9.35 ± 0.5		
**Fenugreek**	12.55 ± 0.61		7.65 ± 0.5		
**Red pepper**	12.65 ± 0.36		7.65 ± 0.5	9.30 ± 0.42	
**Ginger**	12.72 ± 0.3		2.55 ± 0.08	9.20 ± 0.50	10.5
**Turmeric**	17.93 ± 0.28		10.03 ± 0.17		
**Nutmeg**	18.1 ± 0.66		7.65 ± 0.5	18.23 ± 0.55	10.8
**Allspice**	88.81 ± 2.03		96.8 ± 1		161
**Cinnamon**	99.65 ± 3.75	63.43 ± 0.21	35.7 ± 1	45.24 ± 2.41	160
**Clove**	173.7 ± 2.98		237 ± 23		130

The variations in polyphenol contents may be attributed to the low specificity of the Folin-Ciocalteu reagent, which can react with any reducing substance containing hydroxyl groups, not only the phenolic compounds but also certain sugars and proteins [25]. Consequently, the use of the Folin-Ciocalteu reagent provides an approximate estimation of the phenolic content in a sample. Furthermore, the biosynthesis of secondary metabolites, such as polyphenols, can vary during plant development due to the geo-climatic conditions (high temperatures, exposure to the sun, drought, salinity, etc.). As demonstrated by Falleh et al. [26], the phenolic content of a plant in influence by both intrinsic (genetic) and extrinsic factors such as climatic conditions, cultural practices, maturity at harvest, storage conditions and country of origin. Additionally, it is important to consider the impact of the solvent the and the extraction method on polyphenol content. Przygodzka et al. [18] clearly showed the effect of the type of solvent on the extraction of phenolic compounds from spices.

**Fig. 3:**
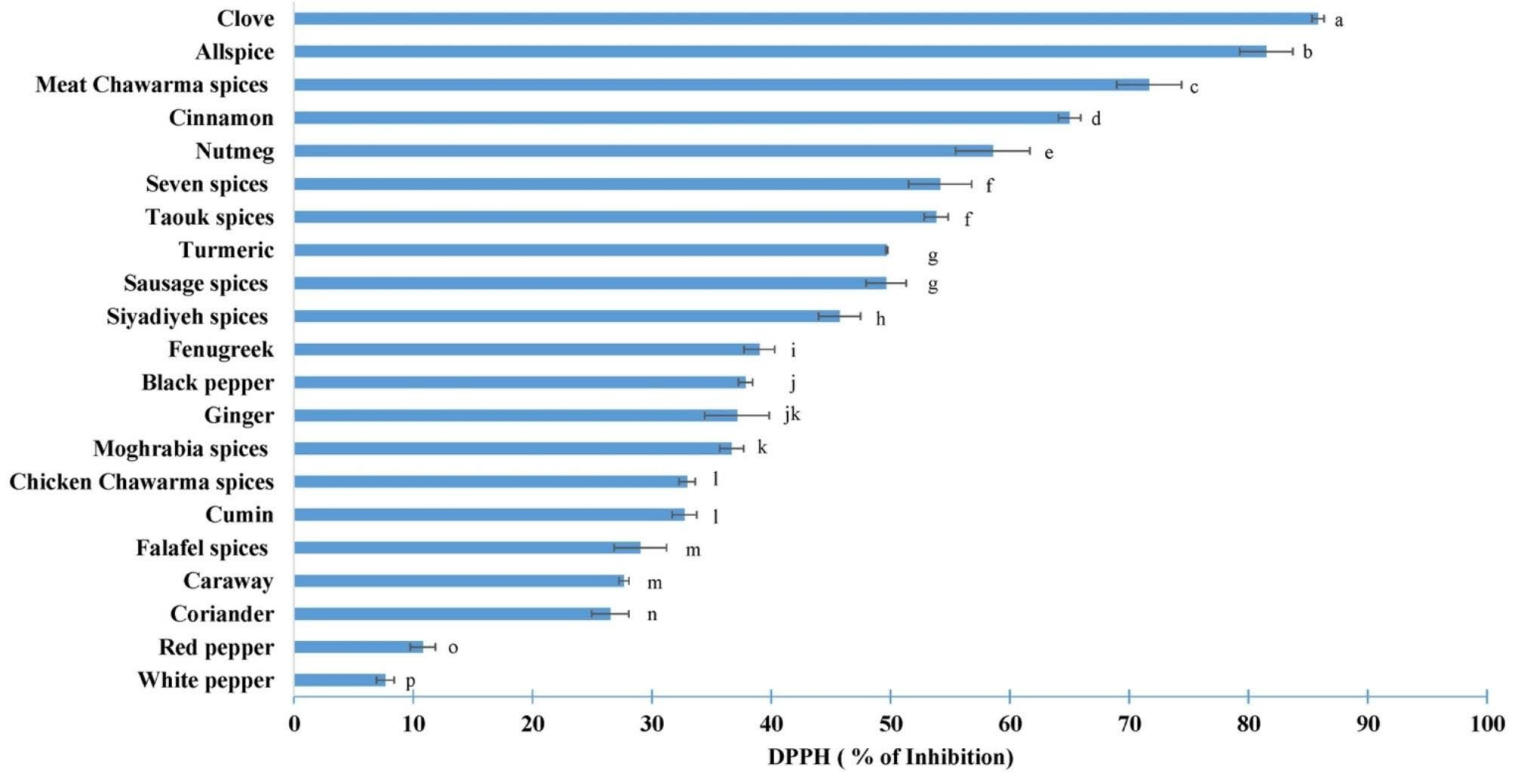
DPPH radical scavenging activities of different spices’ samples. Mean (n = 3). Error bars represent standard deviation. Different letters indicate significant differences at p < 0.05

The antioxidant activity of spice extracts was evaluated using the radical scavenging ability tests (DPPH and ABTS assays). The results of the DPPH assay, as illustrated in Fig. 3, clearly show that the clove extract exhibited the highest DPPH radical scavenging activity, followed by allspice. Moderate antioxidant activities were observed in the case of cinnamon, nutmeg and turmeric extracts. Conversely, spices such as fenugreek, black pepper, ginger, cumin, caraway, coriander, red pepper and white pepper displayed relatively low inhibition percentages, with white pepper exhibiting the lowest among them.

Regarding the spice mixtures, variable percentages of inhibition are observed depending on the ingredients of each mixture. For example, meat chawarma spice exhibited an inhibition percentage of 71.68% ± 2.72, which was relatively close to that of cinnamon and allspice as these were the main components of this mixture. Similarly, falafel spice presented the lowest inhibition percentage (29.04% ± 2.2) due to coriander being the major ingredient in this mixture.

When comparing the present values to those in the literature, similarities are observed. Ali et al. [27] also demonstrated that clove exhibited the highest antioxidant activity among the tested spices in terms of the DPPH test. Shan et al. [23] also demonstrated that clove and cinnamon spices displayed the highest antioxidant activities. In their study, Lu et al. [24] obtained relatively similar values for white pepper (5.35% ± 0.22 vs. 7.66% ± 2.73 in this study) and red pepper (8.31% ± 0.98 vs. 10.8% ± 3.06 in this study). Similarly, the values obtained by Embuscado [28] closely matched those registered in this study for coriander (31% vs. 26.51% ± 1.5), cumin (35.8% vs. 32.73% ± 1.0) and cloves (88.3% vs. 85.84% ± 0.5).

**Fig. 4:**
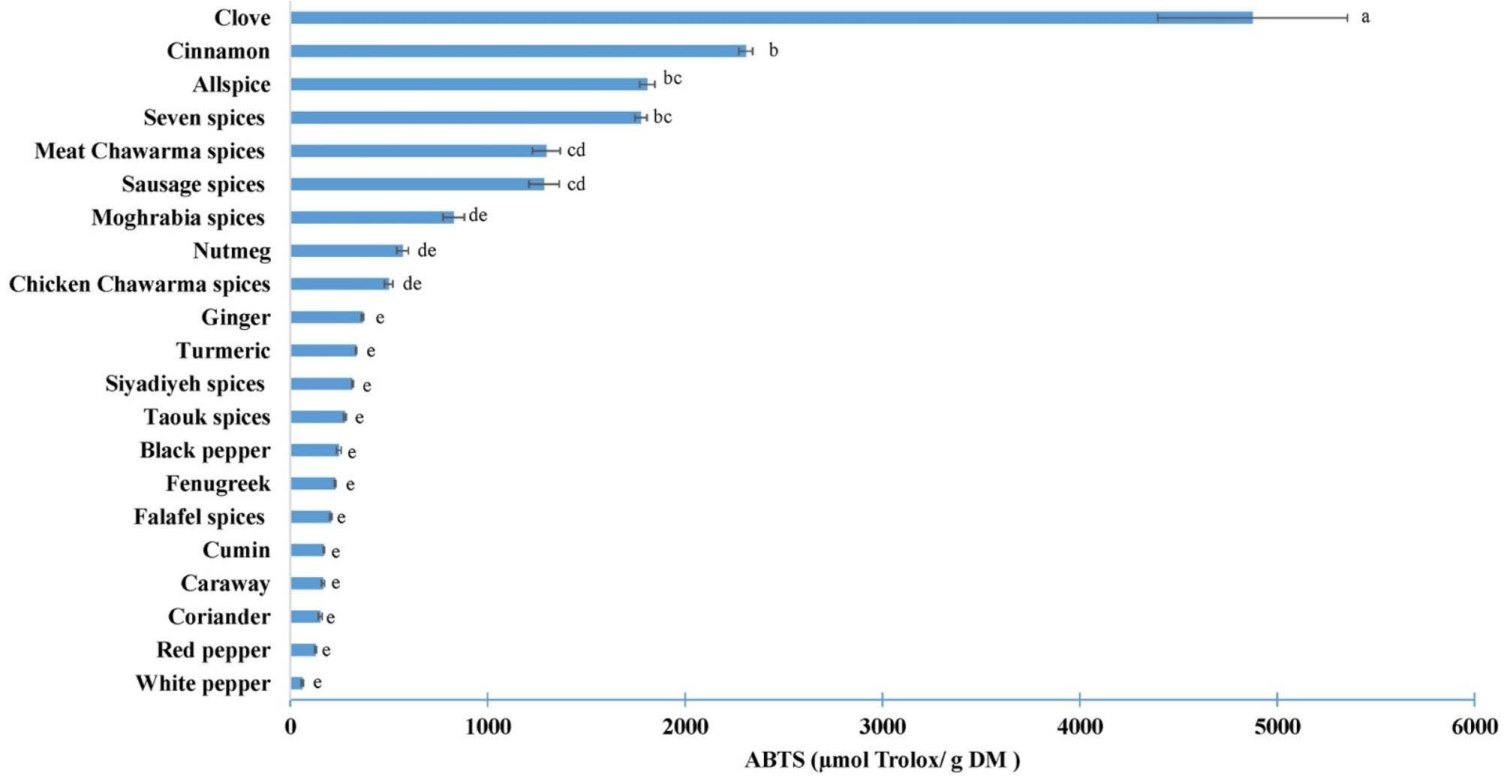
ABTS radical scavenging activities of different spices’ samples. Mean (n = 3). Error bars represent standard deviation. Different letters indicate significant difference at p < 0.05

Furthermore, the antioxidant activity of spices was determined against ABTS radical, as shown in Fig. 4. It is worth noting that the concentration in µmol trolox / g of dry matter is proportional to the antioxidant capacity of the sample. The results in Fig. 4 reveal that the antioxidant activity varies between the different tested spices. The highest antioxidant activity was observed for clove, followed by cinnamon, while the remaining spices, such as coriander, red pepper and white pepper displayed the lowest values.

The results of the spice mixtures presented findings consistent with those obtained in the DPPH test. As previously explained, the antioxidant capacity closely relied on the ingredient’s composition within the mixture. However, with the ABTS assay, the seven spices presented the highest antioxidant activity as opposed to the DPPH assay where meat chawarma yielded the highest results.

In the literature, the ABTS has been widely used to assess the antioxidant activity of spices extracts. For white pepper, values ranged between 12.42 µmol trolox / g DM [24] and 89.7 µmol trolox / g DM [23]. The antioxidant activity of cinnamon varied between 417.9 µmol trolox / g DM [10] and 1119.9 µmol trolox / g DM [18]. Clove consistently exhibited the highest antioxidant activity, ranging from 1018 µmol trolox / g DM [10] to 2071 µmol trolox / g DM [18].

The antioxidant capacity of spices is greatly influenced by the composition of the extracts including factors such as the country of origin, cultural practices and maturity at harvesting, among others. It is also influenced by the test handling conditions, including choice of solvent and polarity, extraction method and time/ temperature couple. These factors may explain the differences observed between the results of the present study and those found in the literature.

Phenolic compounds are recognized for their ability to exhibit antioxidant activity, attributed to their capability to neutralize reactive radical species. This mechanism is linked to the transfer of antioxidant electrons and /or hydrogen atoms to the radicals [29]. The antioxidant activity of phenolic compounds depends upon the arrangement of the functional groups around the basic structure [30]. It is significantly influenced by the number of hydroxyl groups and their position related to the carboxyl group [31]. It is also correlated with the thermodynamic properties of the radical, which influences the stability of the radicals [31]. The antioxidant mechanisms of phenolic compounds, as documented in the literature, can be summarised as follows: (a) free radical scavenging activity, (b) metal ions chelation and reduction, (c) activation of antioxidant enzymes and (d) inhibition of enzymes associated with free-radicals generation [10, 29, 30].

Multiple studies [23, 24, 28] have reported that phenolic compounds in spices contribute to the antioxidant activity. These studies have demonstrated a strong positive linear correlation between the phenolic content and the antioxidant activity of spices. Therefore, the Pearson correlation test was performed to explore the association between the antioxidant activity and the total polyphenol content of the Lebanese spices. The results presented in Table 4 indicated a significant positive correlation between phenolic contents in spices and their free radical scavenging capacity, highlighting the substantial contribution of phenolic compounds to their antioxidant capacity.

**Table 4:** Pearson correlation coefficients

	ABTS (µmol trolox/ g DM)	DPPH (% inhibition)	Total polyphenol (mg GAE/g DM)
ABTS (µmol trolox/ g DM)	**1**		
DPPH (% inhibition)	0.766*	**1**	
Total polyphenol (mg GAE/g DM)	0.788*	0.729*	**1**

Values in bold are different from 0 with a significance level alpha = 0.01, * *p* < 0.01

### Antimicrobial activity

The antimicrobial activity of the 21 selected spices extracts was assessed by determining the minimum inhibitory concentration and the results are presented in Table 5. The MIC values of the spices’ extracts ranged from less than 2.4 µg/ml to 625 µg/ml. Several spices’ extracts exhibited potent antimicrobial activity (≤ 2.4 µg/ml) against all tested bacteria such as cinnamon, turmeric, white pepper, red pepper and allspice. Clove and nutmeg also demonstrated strong antimicrobial activity (≤ 2.4 µg/ml) against *E. coli*, *S. aureus* and *L. monocytogenes*. In contrast, caraway, fenugreek, coriander and ginger displayed mild to low antimicrobial activity with MIC values ranging between 19.5 and 625 µg/ml.

**Table 5:** Antimicrobial activity of Lebanese spices

MIC (µg/mL)											
	Cinnamon	Nutmeg	Coriander	Clove	Cumin	Turmeric	Ginger	White pepper	Fenugreek	Red pepper	
*E. coli* ATCC 8739	< 2.4	< 2.4	78.125	< 2.4	39.06	< 2.4	39.06	< 2.4	625	< 2.4	
* S. aureus* ATCC 25,923	< 2.4	< 2.4	39.06	< 2.4	< 2.4	< 2.4	19.53	< 2.4	39.06	< 2.4	
*Salmonella* Enteritidis	< 2.4	9.765	312.5	4.88	19.5	< 2.4	78.125	< 2.4	312.5	< 2.4	
* L. monocytogenes* ATCC 19,115	< 2.4	< 2.4	39.06	< 2.4	< 2.4	< 2.4	39.06	< 2.4	78.125	< 2.4	
	**Allspice**	**Caraway**	**Black pepper**	**Seven spices**	**Moghrabia**	**Meat Chawarma**	**Sausage**	**Falafel**	**Taouk**	**Siyadiyeh**	**Chicken Chawarma**
*E. coli* ATCC 8739	< 2.4	19.5	< 2.4	< 2.4	< 2.4	< 2.4	78.125	156.25	< 2.4	< 2.4	4.88
* S. aureus* ATCC 25,923	< 2.4	4.88	< 2.4	< 2.4	< 2.4	< 2.4	4.88	78.125	< 2.4	< 2.4	< 2.4
*Salmonella* Enteritidis	< 2.4	39.05	39.05	< 2.4	< 2.4	< 2.4	156.25	312.5	< 2.4	< 2.4	4.88
* L. monocytogenes* ATCC 19,115	< 2.4	19.5	4.88	< 2.4	< 2.4	< 2.4	9.765	39.06	< 2.4	< 2.4	< 2.4

Regarding the antimicrobial activity of the spices mixtures, the results indicated that all the mixtures exhibited high antimicrobial activity except for the falafel and sausage mixture. These mixtures contain a significant amount of spices known for their high antimicrobial activity, such as cinnamon, nutmeg, clove, allspice and white pepper, which collectively contribute to the high antimicrobial effect.

Numerous studies in the literature have demonstrated the antimicrobial activity of spices like cinnamon, clove, allspice, ginger and turmeric against foodborne pathogens bacteria [17, 32–35]. The antibacterial activity of spices is mainly due to the presence of phenolic compounds in their extracts. Additionally, volatile compounds, mainly essential oils, contribute to their antibacterial activity [36]. Compounds such as eugenol, cinnamic aldehyde and curcumin in clove, cinnamon and turmeric have been identified as responsible for antimicrobial activity [37–40]. The hydroxyl groups in the phenolic compounds are responsible for the antimicrobial activity, as they promote the delocalisation of electrons, which then act as proton exchangers and reduce the gradient across the cytoplasmic membrane of bacterial cells leading to the collapse of the proton motive force and contributing to cell death. Furthermore, these hydroxyl groups can bind the active site of enzymes, disrupting the cell metabolism of bacteria [31, 40].

In general, the variation in the antimicrobial activity of spices extracts against bacteria is due to various factors such as the type of microorganisms, the cell structure of gram-negative and gram-positive bacteria, the phytochemistry composition of spice extracts and the extraction conditions.

Various antimicrobial mechanisms by which plants or spices extracts affect microbial cells have been elucidated. These mechanisms are summarised as follows: disruption of cell membranes, increasing permeability and leakage of intracellular material, effects on the proton pump and the ions channels, disruption of enzyme systems, compromise of the genetic material of bacteria, attack on the phospholipid bilayer of the cell membrane, disruption of protein metabolism, anti-quorum sensing effect, decrease of intracellular pH and induction of cell cycle arrest [17, 29]. Pinto et al. [40] stated additional modes of antimicrobial actions of polyphenols, such as modifications to intracellular pH, interference with the ATP-generating system and inhibition of DNA synthesis.

## Conclusion

Phenolic compounds occurring in aromatic plants and spices have been recognised for their antioxidant and antibacterial properties, making them widely utilized for food preservation in the food industries and by various populations for their therapeutic effects. Spices, a rich source of phenolic compounds, play a vital role in shaping the unique flavours of Lebanese cuisine. However, there is a lack of studies assessing the phenolic content and the antimicrobial and antioxidant effects of these spices. Therefore, this study was performed on 21 types of spices used in the Lebanese daily diet.

The results revealed those spices such as cinnamon, clove and allspice extracted by ethanol and water (v/v) are rich in phenolic compounds and exhibit high antioxidant activity. The observed positive correlation between polyphenol content and antioxidant activity underscores the central role played by polyphenols as the key contributors to the antioxidant attributes of Lebanese cuisine spices. Additionally, this study demonstrated the substantial antimicrobial activity of clove, turmeric, clove, allspice, white pepper and red pepper against four major foodborne bacteria suggesting the potential utilization of these spices as natural food preservatives.

## Data Availability

The datasets generated and/or analysed during the current study are included in the manuscript. Moreover, the datasets used to generate figures and results are available from the corresponding author on reasonable request.
